# Association between oral health and dementia in the elderly: a population-based study in Korea

**DOI:** 10.1038/s41598-019-50863-0

**Published:** 2019-10-08

**Authors:** Kyeong Hee Lee, Yoon Young Choi

**Affiliations:** Department of Dental Hygiene, College of Bioecological Health, Shinhan University, Uijeongbu, 11644 Republic of Korea

**Keywords:** Dental epidemiology, Gerodontics, Geriatrics

## Abstract

We have investigated the association of oral health with development of dementia in elderly Koreans. Data for subjects aged ≥65 years who underwent regular National Health Insurance Service check-ups and received dental care in 2017 were analysed. Those treated for dementia in 2017 served as the dementia group and those with no record of dementia in 2002–2017 as the control group. Explanatory variables were sex, age, household income, place of residence, smoking status, alcohol consumption, and presence of diabetes mellitus and hypertension, periodontitis, and removable dentures. Regression analysis showed that dementia was significantly more common in women than in men and in those aged ≥81 years than in those aged 65–70 years. The risk of dementia was highest in the ‘upper-middle’ income group and in the rural population. Smokers and those who consumed alcohol were less likely to develop dementia. Subjects with diabetes were more likely to have dementia than those without it, as were those with hypertension. Dementia was less likely in subjects with periodontitis and more likely in those with removable dentures. Therefore, loss of teeth may contribute to development of dementia.

## Introduction

Dementia is a disease that causes loss of cognitive function and interferes with the ability to perform activities of daily living and to participate in social activity^1^. Although not a disease developed exclusively by the elderly, the most common form is ‘senile dementia’, which is caused by degenerative brain disease, such as Alzheimer’s disease (AD) or vascular dementia^2^.

The most common cause of dementia is AD, which accounts for 60–70% of all dementia cases^3^. According to a UN report released in 2007, it is estimated that one in 85 people will be diagnosed with AD-associated dementia by 2050^4^. However, a study reported in 2014 also suggested that a 20% reduction in the major risk factors for dementia could reduce its incidence by 15.3% by 2050^5^. Therefore, there is a growing need to identify and manage the risk factors associated with dementia. However, despite the number of issues identified and studies related to dementia, there is still uncertainty concerning effective intervention strategies to reduce its prevalence^6^.

Norton *et al*.^5^ reported low educational attainment, smoking, physical inactivity, depression, hypertension in middle-age, diabetes mellitus, and mild obesity as risk factors for dementia. A retrospective cohort study that included 10 years of follow-up and analysis of use of medical services by healthy subjects aged ≥60 years who had undergone health care screening suggested that older age, female sex, eating habit, alcohol consumption, cigarette smoking, obesity, high blood pressure, diabetes mellitus, hypertension, heart disease, stroke, depression, intracranial injury, and mild cognitive impairment are risk factors for dementia^7^.

Oral health has also been reported to be strongly associated with dementia^8–11^. Kusdhany *et al*. emphasized that oral hygiene status is associated with cognitive function^8^. A 4-year prospective Japanese study of 2018 subjects found that tooth loss was a strong risk factor for decreased cognitive function in the elderly^9^. Martande *et al*. compared periodontal health status in patients aged 50–80 years and found that the values for all periodontal variables evaluated were higher in patients with AD than in subjects with normal cognitive function and that periodontal status deteriorated with progression of AD^10^. Cho *et al*. found that patients with natural dentition had better cognitive ability (i.e., a higher Mini-Mental State Examination score) than those with removable dentures^11^. Poor oral health in the elderly has been reported to be strongly associated with dementia^8–11^; however, oral health is still considered to be separate from and less important than systemic health^12^.

Epidemiological data are needed to determine the risk factors for development of dementia. The aims of this study were to confirm the prevalence of dementia and to investigate the relationship between dementia and oral health in elderly Korean individuals using the Korean National Health Insurance Service (NHIS) database, which contains representative health data for all Korean citizens.

## Results

### Overall prevalence of dementia

The overall prevalence of dementia was 5.2%. The prevalence was higher in women than in men (6.4% vs 4.0%; Table 1) and in older subjects regardless of sex (P < 0.001). Dementia was significantly less common in subjects with periodontitis than in those without periodontitis and significantly more common in those with removable dentures (P < 0.001).

**Table 1:** Prevalence of dementia according to demographic characteristics and lifestyle factors.

Characteristic		Male		P-value^†^	Female		P-value^†^
		Dementia	Normal		Dementia	Normal	
All		22,189 (4.0)	529,802 (96.0)		35,088 (6.4)	515,399 (93.6)	
Age, years	65–70	5,268 (1.8)	287,735 (98.2)	<0.001	7,548 (2.7)	276,151 (97.3)	<0.001
	71–75	4,835 (3.9)	117,811 (96.1)		7,208 (6.0)	112,702 (94.0)	
	76–80	7,487 (7.2)	96,612 (92.8)		12,543 (11.4)	97,858 (88.6)	
	≥81	4,599 (14.3)	27,644 (85.7)		7,789 (21.4)	28,688 (78.7)	
Household income^a^	Low	2,912 (3.2)	87,076 (96.8)	<0.001	5,512 (5.6)	93,628 (94.4)	<0.001
	Lower-middle	2,601 (2.8)	90,796 (97.2)		3,706 (6.3)	55,625 (93.8)	
	Middle	2,723 (2.8)	70,803 (96.3)		4,290 (6.4)	62,543 (93.6)	
	Upper-middle	4,638 (4.1)	108,313 (95.9)		6,850 (6.1)	106,445 (94.0)	
	High	9,315 (5.1)	172,814 (94.9)		14,730 (7.0)	197,158 (93.1)	
Residence	Urban	17,666 (3.8)	451,638 (96.2)	<0.001	27,202 (5.9)	436,738 (94.1)	<0.001
	Rural	4,523 (5.5)	78,164 (94.5)		7,886 (9.1)	78,661 (90.9)	
Smoking	Yes	2,464 (2.9)	83,512 (97.1)	<0.001	348 (5.8)	5,629 (94.2)	0.079
	No	19,725 (4.2)	446,290 (95.8)		34,740 (6.4)	509,770 (93.6)	
Alcohol consumption	Yes	3,434 (2.2)	149,583 (97.8)	<0.001	610 (3.4)	17,138 (96.6)	<0.001
	No	18,755 (4.7)	380,219 (95.3)		34,478 (6.5)	498,261 (93.5)	
Diabetes mellitus	Yes	9,665 (5.3)	172,648 (94.7)	<0.001	14,304 (8.4)	156,517 (91.6)	<0.001
	No	12,524 (3.4)	357,154 (96.6)		20,784 (5.5)	358,882 (94.5)	
Hypertension	Yes	15,027 (4.7)	308,332 (95.4)	<0.001	24,799 (7.5)	305,876 (92.5)	<0.001
	No	7,162 (3.1)	221,470 (96.9)		10,289 (4.7)	209,523 (95.3)	
Periodontitis	Yes	1,157 (2.6)	43,012 (97.4)	<0.001	1,793 (4.2)	41,069 (95.8)	<0.001
	No	21,032 (4.1)	486,790 (95.9)		33,295 (6.6)	474,330 (93.4)	
Denture wear	Yes	5,498 (6.6)	78,332 (93.4)	<0.001	9,092 (10.4)	78,383 (89.6)	<0.001
	No	16,691 (3.6)	451,470 (96.4)		25,996 (5.6)	437,016 (94.4)	

^†^P-value calculated by chi-squared test. ^a^Quintiles based on the insurance fee imposed on each household.

The data are presented as n(%).

### Oral health and prevalence of dementia by sex and age

We divided the study participants into 8 groups according to sex and age to minimize bias. The prevalence of dementia according to oral health status is shown for each group in Table 2. The prevalence of dementia in each group according to confounders is shown in Supplementary Tables S1–S4. In all groups, the prevalence of dementia was significantly lower in subjects with periodontitis than in those without it. The prevalence of dementia was significantly higher in the subjects who used a denture (P < 0.001), except in the group of women aged ≥81 years.

**Table 2:** Prevalence of dementia according to oral health status and age category.

Age, years	Oral health		Male		P-value^†^	Female		P-value^†^
			Dementia	Normal		Dementia	Normal	
65–70	Periodontitis	Yes	377 (1.4)	27,161 (98.6)	<0.001	617 (2.3)	25,816 (97.7)	<0.001
		No	4,891 (1.8)	260,574 (98.2)		6,931 (2.7)	250,335 (97.3)	
	Denture wear	Yes	795 (2.5)	30,579 (97.5)	<0.001	989 (3.8)	25,394 (96.3)	<0.001
		No	4,473 (1.7)	257,156 (98.3)		6,559 (2.6)	250,757 (97.5)	
71–75	Periodontitis	Yes	284 (3.1)	8,988 (96.9)	<0.001	425 (4.8)	8,526 (95.3)	<0.001
		No	4,551 (4.0)	108,823 (96.0)		6,783 (6.1)	104,176 (93.9)	
	Denture wear	Yes	982 (5.1)	18,247 (94.9)	<0.001	1,520 (7.7)	18,245 (92.3)	<0.001
		No	3,853 (3.7)	99,564 (96.3)		5,688 (5.7)	94,457 (94.3)	
76–80	Periodontitis	Yes	349 (5.7)	5,751 (94.3)	<0.001	552 (8.8)	5,706 (91.2)	<0.001
		No	7,138 (7.3)	90,861 (92.7)		11,991 (11.5)	92,152 (88.5)	
	Denture wear	Yes	2,060 (9.0)	20,872 (91.0)	<0.001	3,669 (13.1)	24,350 (86.9)	<0.001
		No	5,427 (6.7)	75,740 (93.3)		8,874 (10.8)	73,508 (89.2)	
≥81	Periodontitis	Yes	147 (11.7)	1,112 (88.3)	0.007	199 (16.3)	1,021 (83.7)	<0.001
		No	4,452 (14.4)	26,532 (85.6)		7,590 (21.5)	27,667 (78.5)	
	Denture wear	Yes	1,661 (16.1)	8,634 (83.9)	<0.001	2,914 (21.9)	10,394 (78.1)	0.055
		No	2,938 (13.4)	19,010 (86.6)		4,875 (21.0)	18,294 (79.0)	

^†^P-value calculated by chi-squared test. The data are presented as n(%).

### Association between periodontitis and dementia by sex and age

Table 3 shows the outcome of the logistic regression analyses evaluating the association between periodontitis and dementia in each group. The odds ratio (OR) for model 1 was estimated to be from 0.62 to 0.79 in men and from 0.62 to 0.86 in women (P < 0.01). The OR in model 3, which was adjusted for all confounding factors, was from 0.77 to 0.85 in men and from 0.77 to 0.88 in women, with the ORs in all groups being statistically significant (P < 0.01), except for the group of men aged ≥81 years.

**Table 3:** Association between periodontitis and dementia by sex and age.

Sex	Age, years	Model 1^a^		Model 2^b^		Model 3^c^	
		OR	95% CI	OR	95% CI	OR	95% CI
Male	All	0.62^***^	0.59–0.66	0.79^***^	0.74–0.84	0.79^***^	0.74–0.83
	65–70	0.74^***^	0.67–0.82	0.77^***^	0.69–0.85	0.77^***^	0.69–0.85
	71–75	0.76^***^	0.67–0.85	0.77^***^	0.68–0.87	0.77^***^	0.68–0.87
	76–80	0.77^***^	0.69–0.86	0.80^***^	0.72–0.90	0.80^***^	0.72–0.89
	≥81	0.79^**^	0.66–0.94	0.86	0.72–1.02	0.85	0.71–1.02
Female	All	0.62^***^	0.59–0.65	0.82^***^	0.78–0.86	0.81^***^	0.77–0.85
	65–70	0.86^***^	0.79–0.94	0.89^**^	0.82–0.96	0.88^**^	0.81–0.96
	71–75	0.77^***^	0.69–0.85	0.79^***^	0.72–0.88	0.79^***^	0.71–0.87
	76–80	0.74^***^	0.68–0.81	0.79^***^	0.72–0.86	0.78^***^	0.72–0.86
	≥81	0.71^***^	0.61–0.83	0.77^***^	0.66–0.90	0.77^***^	0.66–0.89

^a^Model 1 was unadjusted; ^b^model 2 was adjusted for age, household income, and residence; ^c^model 3 was adjusted for age, household income, residence, smoking, alcohol consumption, diabetes mellitus, and hypertension. Response variable; dementia (ref. no). ^*^P < 0.05, ^**^P < 0.01, ^***^P < 0.001. CI, confidence interval; OR, odds ratio.

### Association between denture wear and dementia by sex and age

Logistic regression analyses were performed to determine the association between denture use and dementia in each group. In men, the OR was from 1.25 to 1.90 in model 1 and from 1.19 to 1.40 in model 3, with all ORs being statistically significant (P < 0.001; Table 4). The ORs for the 3 groups for women aged 65–70, 71–75, and 75–80 years were from 1.25 to 1.95 in model 1 and from 1.18 to 1.35 in model 3 (P < 0.001); however, the ORs in the group of women aged ≥81 years were not statistically significant.

**Table 4:** Association between denture wear and dementia by sex and age.

Sex	Age, years	Model 1^a^		Model 2^b^		Model 3^c^	
		OR	95% CI	OR	95% CI	OR	95% CI
Male	All	1.90^***^	1.84–1.96	1.32^***^	1.28–1.36	1.32^***^	1.28–1.36
	65–70	1.50^***^	1.39–1.61	1.43^***^	1.32–1.54	1.40^***^	1.30–1.51
	71–75	1.39^***^	1.29–1.49	1.39^***^	1.29–1.49	1.37^***^	1.27–1.47
	76–80	1.38^***^	1.31–1.45	1.33^***^	1.26–1.40	1.34^***^	1.27–1.41
	≥81	1.25^***^	1.17–1.33	1.17^***^	1.10–1.25	1.19^***^	1.11–1.27
Female	All	1.95^***^	1.90–2.00	1.19^***^	1.16–1.22	1.19^***^	1.15–1.22
	65–70	1.49^***^	1.39–1.59	1.39^***^	1.30–1.49	1.35^***^	1.26–1.45
	71–75	1.38^***^	1.31–1.47	1.31^***^	1.24–1.39	1.30^***^	1.22–1.38
	76–80	1.25^***^	1.20–1.30	1.18^***^	1.13–1.23	1.18^***^	1.13–1.23
	≥81	1.05	1.00–1.11	1.01	0.95–1.06	1.01	0.96–1.06

^a^Model 1 was unadjusted; ^b^model 2 was adjusted for age, household income, and residence; ^c^model 3 was adjusted for age, household income, residence, smoking, alcohol consumption, diabetes mellitus, and hypertension. Response variable: dementia (ref. no). *P < 0.05, **P < 0.01, ***P < 0.001. CI, confidence interval; OR, odds ratio.

### Factors affecting the prevalence of dementia

Table 5 shows the results of multiple logistic regression analysis of the explanatory variables, including sex, age, household income, place of residence, smoking, alcohol consumption, presence of diabetes, hypertension, periodontitis, and using removable dentures, as well as of the response variable, i.e., the prevalence of dementia. All explanatory variables were found to have a statistically significant effect on the likelihood of developing dementia (P < 0.001) and were therefore included in the regression model. The fitted regression model was statistically significant (P < 0.001) and the Nagelkerke R^2^ value was 0.109.

**Table 5:** Factors affecting the prevalence of dementia.

Characteristic	B	SE	β	OR	95% CI		χ^2^	P-value^†^
Constant	−4.22	0.02					64596.14	<0.001
Sex (male = 1)	0.36	0.01	0.10	1.43	1.41	1.46	1416.78	<0.001
**Age**, **years (65–70** = **1)**								
71–75	0.76	0.01	0.17	2.13	2.08	2.19	3352.34	<0.001
76–80	1.37	0.01	0.30	3.92	3.83	4.01	12877.58	<0.001
≥81	2.08	0.01	0.28	8.00	7.78	8.22	21987.18	<0.001
**Household income**^**a**^ **(low** = **1)**								
Lower-middle	0.01	0.02	0.00	1.01	0.97	1.04	0.10	0.748
Middle	0.06	0.02	0.01	1.07	1.03	1.10	14.08	<0.001
Upper-middle	0.08	0.02	0.02	1.09	1.05	1.12	30.08	<0.001
High	0.07	0.01	0.02	1.08	1.05	1.10	28.95	<0.001
Residence (urban = 1)	0.30	0.01	0.06	1.35	1.32	1.37	746.63	<0.001
Smoking (no = 1)	−0.08	0.02	−0.01	0.92	0.88	0.96	16.39	<0.001
Alcohol consumption (no = 1)	−0.47	0.02	−0.09	0.63	0.60	0.65	716.26	<0.001
Diabetes mellitus (no = 1)	0.38	0.01	0.10	1.46	1.43	1.49	1722.26	<0.001
Hypertension (no = 1)	0.16	0.01	0.04	1.17	1.15	1.19	258.47	<0.001
Periodontitis (no = 1)	−0.22	0.02	−0.03	0.80	0.77	0.83	126.75	<0.001
Denture (no = 1)	0.23	0.01	0.05	1.26	1.24	1.29	490.23	<0.001

χ^2^ = 40963.03, P < 0.001, Nagelkerke R^2^ = 0.109. ^†^P-value calculated by logistic regression analysis. ^a^Quintiles based on the insurance fee imposed on each household. B, unstandardized beta; SE, standardized error; β, standardized beta; OR, odds ratio; CI, confidence interval.

Dementia was more common in women than in men (OR 1.43; P < 0.001) and more common in those aged ≥81 years than in those aged 65–70 years (OR 8.00; P < 0.001). Subjects with an ‘upper-middle’ household income were more likely to have dementia (OR 1.09; P < 0.001), as were residents of rural areas when compared with those from urban areas (OR 1.35; P < 0.001). Dementia was less common in smokers than in non-smokers (OR 0.92; P < 0.001) and in those who consumed alcohol than in those who did not (OR 0.63; P < 0.001). Subjects with diabetes were more likely to have dementia than those who did not (OR 1.46; P < 0.001), as were those with hypertension (OR 1.17; P < 0.001). Dementia was less common in subjects with periodontitis (OR 0.80; P < 0.001) but was more common in those with removable dentures (OR 1.26; P < 0.001).

## Discussion

Dementia is a disease that progresses slowly over a period of 5–20 years after onset, and prevention is most important^13^. Health determinants that act as risk factors for dementia, including sociodemographic characteristics, health behaviours, and systemic diseases, should be recognized in an effort to prevent the disease^6^. This study investigated a large number of variables, including sex, age, household income, and place of residence as sociodemographic characteristics, smoking and drinking as health behaviours, and diabetes and hypertension as systemic diseases.

Advanced age is the strongest risk factor for dementia. In a previous study^14^, the prevalence of dementia at the age of 65–69 years was 1.3%, with a two-fold increase every 5 years; thereafter, the prevalence in the population aged ≥85 years has been reported to be 30.5%^14^. In the present study, the prevalence of dementia was 14.3% and 21.4% for men and women in the group aged ≥81 years and 1.8% and 2.7% in the group aged 65–70 years, which is consistent with a previous report^14^. Moreover, in a study reported in 2018, the risk of dementia was significantly higher in women than in men, in smokers than in non-smokers, and in those who consumed alcohol than in those who did not^15^. Furthermore, Kim^16^ reported that female sex, age >70 years, a low educational level, and alcohol abuse were risk factors for cognitive impairment. The results of multiple logistic regression analysis in the present study also showed that women were more likely to develop dementia than men (OR 1.43; P < 0.001) while subjects aged ≥81 years were more likely to have dementia than those aged 65–70 years (OR 8.00; P < 0.001). However, in this study, smokers were less likely to have dementia than non-smokers and the same was true for alcohol consumption. These findings may reflect the fact that subjects who had drinking or smoking habits may have quit them after the onset of dementia either on their own or under the care of nursing homes and caregivers. Therefore, it was difficult to determine the effects of smoking and alcohol consumption on dementia in this study.

Those with an ‘upper-middle’ household income were more likely to develop dementia (OR 1.09; P < 0.001) in this study. This finding is different from that in the study by Ribeiro *et al*., who showed that patients with AD had a lower average monthly income^17^, and from that in a study of urban-dwelling African-Americans that investigated factors influencing cognitive function, which showed that a low income level was associated with poor cognitive function^18^. Further studies are needed to verify these findings.

Rural residents were more likely to have dementia than their urban counterparts (OR 1.35; P < 0.001). This finding may reflect the effects of social isolation on development of dementia found by Lee^19^ in a study that investigated dementia and social isolation among the rural elderly in Korea. Hence, elderly individuals residing in rural areas may have an increased risk of dementia because of their limited social networks when compared with elderly residents of urban areas.

Hypertension and diabetes mellitus are known to be risk factors for dementia^20,21^, with an increase in the prevalence of the disease as age increases. Being elderly is an uncontrollable risk factor for dementia; however, hypertension and diabetes mellitus are controllable risk factors^22^. In this study, patients with diabetes mellitus were also more likely to have dementia than healthy elderly subjects (OR 1.46; P < 0.001) and the same was true for patients with hypertension (OR 1.17; P < 0.001). Therefore, continuous health care before the onset of old age to prevent chronic illnesses such as hypertension and diabetes mellitus may help to lower the risk of dementia.

Periodontitis is a chronic inflammatory disease that has been identified as a potential risk factor for a variety of systemic disorders, including cardiovascular disease, diabetes mellitus, hypertension, respiratory disease, osteoporosis, metabolic syndrome, and rheumatoid arthritis^23,24^. An association between periodontitis and dementia has also been suggested^25–28^. The direct and indirect causes of dementia are thought to involve inflammatory reactions, damage caused by oxygen-derived free radicals that damage cells, and toxic substances; periodontitis, a chronic inflammatory disease, is thought to impair cognition via this mechanism^25,26^. Periodontitis has been reported to be a risk factor for the onset and progression of AD^27^ and has been suggested to exacerbate inflammatory conditions in the elderly, thereby accelerating the progression of neurodegenerative diseases^28^. Furthermore, a longitudinal study investigating the risk of dementia following chronic periodontitis in Koreans by Cox proportional hazards regression recently suggested that chronic periodontitis could increase the risk of dementia^29^. However, in the present study, subjects with periodontitis were less likely to have dementia (OR 0.80; P < 0.001), and the prevalence of dementia was lower in subjects with periodontitis even when analysed by sex and age. We expected that the elderly with dementia would have more tooth loss than their counterparts without dementia, as previously reported^30–32^, and in this study considered that tooth loss would reduce the need for treatment of periodontitis and decrease its prevalence. Using the NHIS data, we determined the presence of chronic disease by using a diagnostic code for medical and dental care^33^, but the true prevalence of periodontal disease might not have been reflected if individuals with a given disease stopped treatment. Therefore, there is a possibility of underestimation of prevalence. However, it is also possible that the prevalence of periodontitis is low in patients with dementia. This possibility needs investigation in the future.

Subjects using a removable partial denture or full dentures are very likely to have lost a high number of teeth. Our results showed that patients with removable dentures were more likely to have dementia (OR 1.26; P < 0.001) regardless of sex and age. Syrjälä *et al*.^30^ reported that subjects with dementia are likely to be edentulous (OR 5.2; 95% CI 1.0–26.6). Moreover, Cho^31^ reported that for persons with 0–10 residual teeth, the OR for probability of dementia was 3.527 (95% CI 1.382–8.997) based on the Mini-Mental State Examination score in individuals with a residual tooth number of ≥21. Other studies have also found that individuals with cognitive impairment have fewer remaining teeth than those without cognitive impairment^32^. However, in a study examining the relationship between dental health and dementia in the elderly using the Korean Dementia Screening Questionnaire^3,34^, neither the number of functional teeth, including implants and fixed prostheses, nor use of dentures had a significant effect on the risk of dementia, unlike in previous studies.

This study has several limitations. It is difficult to infer a causal relationship between dementia and the risk factors for dementia using a cross-sectional study design. Furthermore, the NHIS data do not reveal the number of teeth lost, so there is a limit to estimation of tooth loss when using data for subjects treated with removable dentures. However, despite these limitations, it was possible to control recall bias, which is common in questionnaire-based research, because of use of NHIS data, which is meaningful for confirmation of the effect of dental health on dementia in the elderly. Therefore, we are planning a longitudinal cohort study to investigate the causal relationship between dementia and oral health. In the meantime, we hope that our findings will contribute to the development of health promotion programs and effective strategies for prevention of dementia.

## Methods

### Participants

The study protocol was approved by the Institutional Review Board of Shinhan University (SHIRB-201806-HR-082-01) and conducted following the guidelines of the Declaration of Helsinki. The requirement for written informed consent was waived because the study used the NHIS database, which contains only anonymised secondary data.

**Figure 1:**
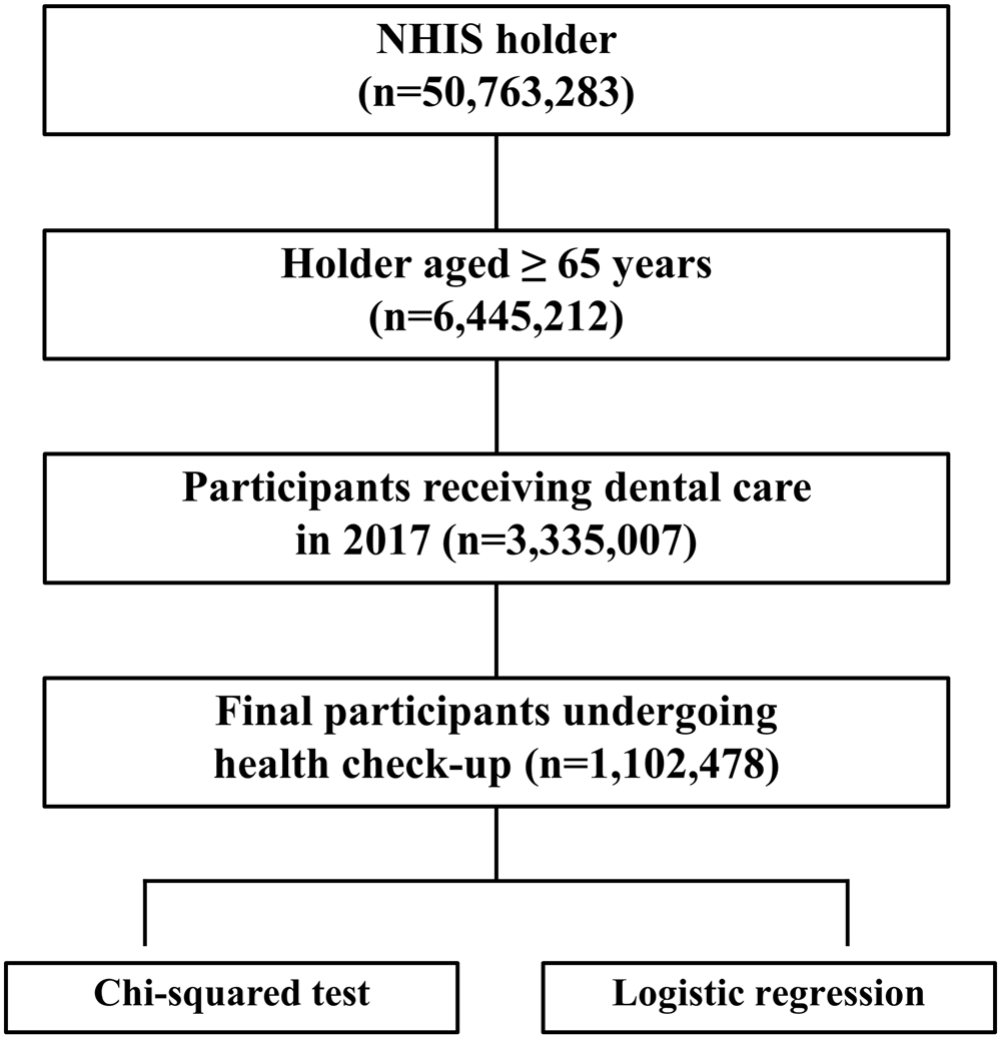
Flow of participants through the study. NHIS, National Health Insurance Service.

Almost all Korean citizens (approximately 98%) are covered by Korean national health insurance, and their sociodemographic and medical records, prescription medication history, and history of medical and dental health screening are stored in the NHIS database. This database contains data for patients aged ≥65 years who received dental treatment in 2017, who was an eligibility criterion for this study. Patients who also underwent regular health check-ups performed by the NHIS were selected as the final subjects for this study (Fig. 1).

### Study variables

Subjects who had a history of treatment for dementia in their medical records for 2017 were defined as patients with dementia and those with no record of dementia treatment between 2002 (the year medical records started) and 2017 were classified as normal subjects. The diagnostic code for dementia was based on the International Classification of Diseases 10^th^ (ICD-10); dementia was determined by F00 (Dementia in Alzheimer’s disease), F01 (Vascular dementia), F02 (Dementia in other diseases classified elsewhere), F03 (Unspecified dementia), and F05.1 (Delirium superimposed on dementia).

The explanatory variables used in this study were sex, age, household income, place of residence, smoking and drinking status, presence of diabetes mellitus and hypertension, periodontitis, and wearing of removable dentures. Age was classified as 65–70, 71–75, 76–80, and ≥81 years. Household income was classified into quintiles (low, lower-middle, middle, upper-middle, high) based on the cost of the health insurance paid, and place of residence was classified as urban or rural. Furthermore, current smoking status and drinking status were examined based on the responses to the NHIS health screening questionnaire. Ex-smokers were classified as non-smokers, and current drinking status was judged based on drinking alcohol more than once a week.

Diabetes, hypertension, periodontitis, and wearing a denture were assessed according to the medical records of dental and medical institutions. First, based on the ICD-10, we looked at the medical records of the subjects to see if there were any records corresponding to the diagnostic code for diabetes mellitus (E10–E14) or hypertension (I10–I15). The definition of periodontitis was modified for the purposes of this study with reference to a previous definition^35^. Patients who had had periodontal treatment (U1010; subgingival curettage, U1051–1052; periodontal flap operation, U1060; root conditioning, U1071–1072; bone graft for alveolar bone defects, U1081–1083; guided tissue regeneration) with a diagnostic code for periodontitis (K05.2; acute periodontitis, K05.3; chronic periodontitis, K05.4; other periodontal disease, K05.6; periodontal disease unspecified) were defined as patients with periodontitis. Furthermore, subjects were deemed to use a removable denture if there were any medical records including the treatment code related to the manufacturing process of a removable partial denture (UA301–UA359, UA401–UA419), the treatment code related to the manufacturing process for a full denture (UA101-UA149, UA201-UA209, UA501-UA549), and the treatment code related to the denture repair (U1511-U1542).

### Statistical analysis

The differences in prevalence of dementia according to subject characteristics were analysed using the chi-square test. We also divided the study participants into 8 groups according to sex and age and analysed the prevalence of dementia according to the oral health of each group. Furthermore, multiple regression analyses were conducted to determine the associations of periodontitis and denture wear with dementia in each group, with sequential inclusion of confounding factors in the models 1–3. Finally, multiple logistic regression analysis was performed with all confounding factors using periodontitis and denture wear as explanatory variables and dementia as the response variable. All statistical analyses were performed using SAS software (version 9.4; SAS Institute Inc., Cary, NC, USA). A P-value of 0.05 was considered statistically significant.

## Supplementary information

Supplementary tables

## Data Availability

The data that support the findings of this study are available from the National Health Insurance Service–National Sample Cohort but restrictions apply to the availability of these data, which were used under license for the current study, and so are not publicly available.
