# Supplementary material for: Association between oral health and dementia in the elderly: a population-based study in Korea
Source: Sci Rep. 2019 Oct 8;9:14407. doi: 10.1038/s41598-019-50863-0 (PMC6783535; doi:10.1038/s41598-019-50863-0)
Supplement: Supplementary file 1 — Supplementary tables [file 41598_2019_50863_MOESM1_ESM.pdf]

Association between oral health and dementia in the elderly: a population-based study in Korea

Kyeong Hee Lee, PhD[1], Yoon Young Choi, PhD[1]*

[1]Department of Dental Hygiene, College of Bioecological Health, Shinhan University, Uijeongbu 11644, Republic of Korea

***Corresponding author:** E-mail: dencyy@naver.com (YYC)

Table S1. Prevalence of dementia according to confounding variables in study participants aged 65–70 years

Characteristic		Male		P-value†	Female		P-value†
		Dementia	Normal		Dementia	Normal	
Household income[a]	Low	888 (1.8)	49,822 (98.3)	<0.001	1,332 (2.3)	57,894 (97.8)	<0.001
	Lower-middle	874 (1.4)	60,877 (98.6)		927 (2.8)	32,698 (97.2)	
	Middle	786 (1.8)	43,821 (98.2)		1,009 (2.9)	34,249 (97.1)	
	Upper-middle	1,294 (2.1)	60,811 (97.9)		1,757 (2.8)	60,840 (97.2)	
	High	1,426 (1.9)	72,404 (98.1)		2,523 (2.7)	90,470 (97.3)	
Residence	Urban	4,368 (1.7)	248,475 (98.3)	<0.001	6,271 (2.6)	238,985 (97.4)	<0.001
	Rural	900 (2.2)	39,260 (97.8)		1,277 (3.3)	37,166 (96.7)	
Smoking	Yes	826 (1.5)	54,194 (98.5)	<0.001	93 (2.6)	3,479 (97.4)	0.831
	No	4,442 (1.9)	233,541 (98.1)		7,455 (2.7)	272,672 (97.3)	
Alcohol consumption	Yes	1,151 (1.2)	95,608 (98.8)	<0.001	218 (1.8)	12,260 (98.3)	<0.001
	No	4,117 (2.1)	192,127 (97.9)		7,330 (2.7)	263,891 (97.3)	
Diabetes mellitus	Yes	2,362 (2.5)	90,906 (97.5)	<0.001	2,984 (3.8)	76,262 (96.2)	<0.001
	No	2,906 (1.5)	196,829 (98.6)		4,564 (2.2)	199,889 (97.8)	
Hypertension	Yes	3,396 (2.1)	156,440 (97.9)	<0.001	4,560 (3.1)	142,259 (96.9)	<0.001
	No	1,872 (1.4)	131,295 (98.6)		2,988 (2.2)	133,892 (97.8)	

†P-value calculated using the chi-squared test. [a]Quintiles based on the insurance fee imposed on each household.

Table S2. Prevalence of dementia according to confounding variables in study participants aged 71–75 years

Characteristic		Male		P-value†	Female		P-value†
		Dementia	Normal		Dementia	Normal	
Household income[a]	Low	703 (3.1)	21,793 (96.9)	<0.001	1,033 (5.2)	18,988 (94.8)	<0.001
	Lower-middle	603 (3.2)	18,450 (96.8)		725 (6.5)	10,417 (93.5)	
	Middle	584 (4.1)	13,589 (95.9)		900 (6.4)	13,207 (93.6)	
	Upper-middle	1,104 (4.4)	23,767 (95.6)		1,489 (6.2)	22,578 (93.8)	
	High	1,841 (4.4)	40,212 (95.6)		3,061 (6.1)	47,512 (94.0)	
Residence	Urban	3,978 (3.8)	100,757 (96.2)	<0.001	5,702 (5.7)	95,285 (94.4)	<0.001
	Rural	857 (4.8)	17,054 (95.2)		1,506 (8.0)	17,417 (92.0)	
Smoking	Yes	609 (3.5)	16,767 (96.5)	0.001	73 (6.6)	1,028 (93.4)	0.385
	No	4,226 (4.0)	101,044 (96.0)		7,135 (6.0)	111,674 (94.0)	
Alcohol consumption	Yes	840 (2.7)	30,493 (97.3)	<0.001	117 (4.0)	2,786 (96.0)	<0.001
	No	3,995 (4.4)	87,318 (95.6)		7,091 (6.1)	109,916 (93.9)	
Diabetes mellitus	Yes	2,300 (5.5)	39,427 (94.5)	<0.001	3,028 (7.6)	36,994 (92.4)	<0.001
	No	2,535 (3.1)	78,384 (96.9)		4,180 (5.2)	75,708 (94.8)	
Hypertension	Yes	3,192 (4.3)	70,492 (95.7)	<0.001	4,964 (6.5)	71,376 (93.5)	<0.001
	No	1,643 (3.4)	47,319 (96.6)		2,244 (5.2)	41,326 (94.9)	

†P-value calculated using the chi-squared test. [a]Quintiles based on the insurance fee imposed on each household.

Table S3. Prevalence of dementia according to confounding variables in study participants aged 76–80 years

Characteristic		Male		P-value[†]	Female		P-value[†]
		Dementia	Normal		Dementia	Normal	
Household income[a]	Low	790 (6.0)	12,460 (94.0)	<0.001	1,703 (12.1)	12,403 (87.9)	0.020
	Lower-middle	687 (7.1)	8,936 (92.9)		1,248 (11.7)	9,428 (88.3)	
	Middle	866 (7.7)	10,432 (92.3)		1,433 (11.1)	11,457 (88.9)	
	Upper-middle	1,465 (7.2)	18,984 (92.8)		2,319 (11.4)	17,972 (88.6)	
	High	3,679 (7.4)	45,800 (92.6)		5,840 (11.1)	46,598 (88.9)	
Residence	Urban	5,889 (6.9)	80,000 (93.1)	<0.001	9,509 (10.6)	79,935 (89.4)	<0.001
	Rural	1,598 (8.8)	16,612 (91.2)		3,034 (14.5)	17,923 (85.5)	
Smoking	Yes	762 (6.8)	10,521 (93.3)	0.056	122 (12.4)	860 (87.6)	0.292
	No	6,725 (7.3)	86,091 (92.8)		12,421 (11.4)	96,998 (88.7)	
Alcohol consumption	Yes	1,007 (4.9)	19,476 (95.1)	<0.001	187 (19.98)	1,687 (90.02)	0.057
	No	6,480 (7.8)	77,136 (92.3)		12,356 (11.4)	96,171 (88.6)	
Diabetes mellitus	Yes	3,290 (9.0)	33,244 (91.0)	<0.001	5,344 (13.6)	33,922 (86.4)	<0.001
	No	4,197 (6.2)	63,368 (93.8)		7,199 (10.1)	63,936 (89.9)	
Hypertension	Yes	5,144 (7.6)	62,601 (92.4)	<0.001	9,274 (11.7)	69,937 (88.3)	<0.001
	No	2,343 (6.4)	34,011 (93.6)		3,269 (10.5)	27,921 (89.5)	

[†]P-value calculated using the chi-squared test. [a]Quintiles based on the insurance fee imposed on each household.

Table S4. Prevalence of dementia according to confounding variables in study participants aged ≥81 years

Characteristic		Male		P-value†	Female		P-value†
		Dementia	Normal		Dementia	Normal	
Household income[a]	Low	531 (15.0)	3,001 (85.0)	0.597	1,444 (25.0)	4,343 (75.1)	<0.001
	Lower-middle	437 (14.7)	2,533 (85.3)		806 (20.7)	3,082 (79.3)	
	Middle	487 (14.1)	2,961 (85.9)		948 (20.7)	3,630 (79.3)	
	Upper-middle	775 (14.0)	4,751 (86.0)		1,285 (20.3)	5,055 (79.7)	
	High	2,369 (14.1)	14,398 (85.9)		3,306 (20.8)	12,578 (79.2)	
Residence	Urban	3,431 (13.3)	22,406 (86.7)	<0.001	5,720 (20.3)	22,533 (79.8)	<0.001
	Rural	1,168 (18.2)	5,238 (81.8)		2,069 (25.2)	6,155 (74.8)	
Smoking	Yes	267 (11.6)	2,030 (88.4)	<0.001	60 (18.6)	262 (81.4)	0.232
	No	4,332 (14.5)	25,614 (85.5)		7,729 (21.4)	28,426 (78.6)	
Alcohol consumption	Yes	436 (9.8)	4,006 (90.2)	<0.001	88 (17.9)	405 (82.2)	0.056
	No	4,163 (15.0)	23,638 (85.0)		7,701 (21.4)	28,283 (78.6)	
Diabetes mellitus	Yes	1,713 (15.9)	9,071 (84.1)	<0.001	2,948 (24.0)	9,339 (76.0)	<0.001
	No	2,886 (13.5)	18,573 (86.6)		4,841 (20.0)	19,349 (80.0)	
Hypertension	Yes	3,295 (14.9)	18,799 (85.1)	<0.001	6,001 (21.2)	22,304 (78.8)	0.187
	No	1,304 (12.9)	8,845 (87.2)		1,788 (21.9)	6,384 (78.1)	

†P-value calculated using the chi-squared test. [a]Quintiles based on the insurance fee imposed on each household.
